# National Trends in the Incidence of Sporadic Malignant Colorectal Polyps in Young Patients (20–49 Years): An 18-Year SEER Database Analysis

**DOI:** 10.3390/medicina60040673

**Published:** 2024-04-21

**Authors:** Mark M. Aloysius, Tejas Nikumbh, Lekha Yadukumar, Udit Asija, Niraj J. Shah, Ganesh Aswath, Savio John, Hemant Goyal

**Affiliations:** 1 Division of Gastroenterology, Department of Medicine, State University of New York Upstate Syracuse, New York, NY 13210, USA; vamedicalresident@gmail.com (M.M.A.);; 2 Department of Internal Medicine, The Wright Center for Graduate Medical Education, Scranton, PA 18505, USA; yadukumarl@thewrightcenter.org (L.Y.); asijau@thewrightcenter.org (U.A.); 3 Division of Gastroenterology, University of Mississippi Medical Center, Jackson, MS 39216, USA; 4 Advanced Endoscopy, Borland Groover Owntown Office, Jacksonville, FL 32207, USA

**Keywords:** young-onset colorectal cancer, sporadic malignant polyps, adenomatous adenomas, malignant tubulovillous adenomas, SEER database

## Abstract

*Background and Objectives*: Conflicting guidelines exist for initiating average-risk colorectal cancer screening at the age of 45 years. The United States Preventive Services Task Force (USPSTF) changed its guidelines in 2021 to recommend initiating screening at 45 years due to an increasing incidence of young-onset colorectal cancer. However, the American College of Physicians (ACP) recently recommended not screening average-risk individuals between 45 and 49 years old. We aim to study the national trends in the incidence of sporadic malignant polyps (SMP) in patients from 20 to 49 years old. *Materials and Methods*: We analyzed the Surveillance, Epidemiology, and End Results database (2000–2017) on patients aged 20–49 years who underwent diagnostic colonoscopy with at least a single malignant sporadic colorectal polyp. *Results*: Of the 10,742 patients diagnosed with SMP, 42.9% were female. The mean age of incidence was 43.07 years (42.91–43.23, 95% CI). Approximately 50% of malignant polyps were diagnosed between 45 and 49 years of age, followed by 25–30% between 40 and 45. There was an upward trend in malignant polyps, with a decreased incidence of malignant villous adenomas and a rise in malignant adenomas and tubulovillous adenomas. *Conclusions*: Our findings suggest that almost half of the SMPs under 50 years occurred in individuals under age 45, younger than the current screening threshold recommended by the ACP. There has been an upward trend in malignant polyps in the last two decades. This reflects changes in tumor biology, and necessitates further research and support in the USPSTF guidelines to start screening at the age of 45 years.

## 1. Introduction

Colorectal carcinoma (CRC) is the third most common cancer worldwide and the second most common cause of cancer death. More than 1.9 million new colorectal cancer cases and 935,000 deaths were estimated to occur in 2020, representing about 1 in 10 cancer cases and deaths [1]. CRC is commonly diagnosed in older people, and the median age of diagnosis is 67 years [2]. Early-onset CRC, also known as young-onset colorectal carcinoma (YoCRC), is defined as CRC diagnosed in individuals younger than the age of 50 years who did not previously meet the traditional age criteria for average-risk screening in the United States [3,4]. In 2023, approximately 153,020 people will be diagnosed with CRC, and 52,550 will die from the disease, with 19,550 cases and 3750 deaths occurring in people under the age of 50 [5]. Over the last ten years, the incidence of CRC has been steadily increasing, and there is an increased annual incidence of YoCRC. The incidence of YoCRC has nearly doubled since 1990, with an increase of 2% per year, mainly in Western countries [6,7,8]. Based on current trends, it is anticipated that the incidence rates of cancers of the colon and rectum will rise by 90% and 124.2%, respectively, among people in the 20–34 age group, while they will increase by 27.7% and 46.0%, respectively, among patients in the 35–39 age group [6,9]. YoCRC is now the second and fourth most common cause of cancer in men and women under 50 years in the US. The death rate from YoCRC has been progressively increasing, with an estimated 12 deaths per 100,000 as of 2019 [10].

Young-onset colorectal cancer is associated with a number of risk factors, such as obesity, processed meat consumption, alcohol intake, family history, inflammatory bowel disease, genetic predisposition syndromes, and disruption of the gut microbiome [3,11]. Despite one-third of YoCRC being associated with familial risk factors, the majority of YoCRCs do not have associated hereditary syndromes and are linked with microsatellite instability. YoCRCs are more likely to present as advanced-stage III/IV carcinomas, and more frequently display aggressive histological characteristics such as poor differentiation and perineural and blood vessel invasion [12,13,14]. Patients with YoCRC also have a significantly longer median time to diagnosis, symptom duration, and time of evaluation. The time to diagnosis is 1.4 times longer for younger than older patients [14]. The definitive pathogenesis and molecular profile of YoCRC are not well understood, and there are few studies that have addressed this.

The increase in the incidence of YoCRC, together with the significant proportion of cases with sporadic characteristics within this subgroup of CRC, have led multiple societies to recommend starting regular screening by a stool-based test or colonoscopy at age 45 for people at an average risk for CRC. However, conflicting guidelines exist in terms of initiating average-risk colorectal cancer screening at age 45 years. The US Preventive Services Task Force changed its guidelines in 2021 to recommend initiating screening at 45 years due to the increasing incidence of young-onset colorectal cancer [15]. However, the American College of Physicians (ACP) recently recommended not to screen average-risk individuals between 45 and 49 years [16]. The American Cancer Society (ACS) published recommendations for CRC screening for average-risk adults starting at age 45 in May 2018 [17]. The US Preventive Services Task Force (USPSTF) expanded its recommendations for CRC screening guidelines in 2021 to include adults aged 45–49 [15,18]. This was followed by the American College of Gastroenterology (ACG) amending their CRC screening guidelines to begin from 45 years for average-risk individuals [19].

The initiation of screening at age 45 instead of 50 years added 19 million average-risk people to the screening pool and dropped national CRC screening rates for those 50 and older from 68% to 59% [5]. No specific changes in screening recommendations were made for people at higher-than-average risk by the ACS, USPSTF, or ACG. The USPSTF recommends beginning screening for those with a family history of CRC 10 years before the age of the youngest affected relative’s diagnosis or age 40, whichever is earlier [20]. The NCCN guidelines recommend genetic screening for patients with young-onset CRC [21]. It is of value to note that only half of young-onset CRC patients with germline mutations have a history of CRC in a first-degree relative, despite the fact that family history is frequently used to screen for elevated CRC risk [22].

Owing to the lack of screening in younger patients, the incidence of YoCRC reflects diagnostically detected CRCs in symptomatic patients or inherently high-risk patients. Data about the new case trends in sporadic malignant polyps (SMP) in younger patients are limited, given the lack of established screening in those at an average risk of CRC under the age of 45. The lack of uniformity in guidelines prevents definitive management and screening in the 45 to 49 years age group population. Hence, we aimed to study the trends in new cases of sporadic malignant polyps in patients between 20 and 49 years of age over an 18-year period from the National Cancer Institute’s SEER database.

## 2. Materials and Methods

### 2.1. Study Design

The Surveillance, Epidemiology, and End Results (SEER) Program of the National Cancer Institute (NCI) is an authoritative source of information on cancer incidence and survival in the United States (U.S.). The SEER database contains cancer incidences dating back four decades, from 1975 [23]. It was launched on 1 January 1973 as a part of the National Cancer Act. Initially, 7 registries (SEER 7) with epidemiologically significant populations that consisted of racial and ethnic minorities were included, and this was gradually enlarged to the current 22 cancer registries (SEER 22). SEER collects demographic, clinical, and outcome information on all cancers diagnosed in representative geographic regions and subpopulations. Data are obtained for all primary invasive malignancies and some additional diagnoses, such as in situ carcinomas, and include the date of diagnosis, as well as demographic data such as age, gender, race/ethnicity, and county of residence [24]. Appropriate use of the SEER database can ensure that the correct research conclusions are drawn and maximize the benefits for clinicians and patients [25].

SEER is widely regarded as the gold standard for data quality in US and international cancer registries. For cancer registries worldwide, the SEER Program serves as a model due to its emphasis on quality control from the program’s beginning, its long-standing commitment to representing all population segments, and its recent success in funding research advancements. Contractual arrangements with regional registries ensure quality, and SEER standards must be met before data are transferred [24]. Originally, there were only 9 tumor registries, and now there are 22 US geographic areas participating in the SEER program. Recently, the SEER Program has been moving toward more automation to improve its consistency and reduce delays in cancer reporting. It currently collects and publishes cancer incidence and survival data from population-based cancer registries, covering approximately 41.9 percent of the U.S. population as per the 2020 census, and has the largest geographic coverage available for survival. SEER 17 (accessed in November 2022) includes 17 cancer registries that collect cancer incidence data from various geographic regions of the U.S. SEER 17 contains 1 record for each of 9,208,295 tumors.

The American Community Survey (ACS) is an ongoing community-based survey that has been conducted by the US Census Bureau since 2005. The ACS is the primary source of high-resolution geographic data on the U.S. population. It provides vital demographic, housing, and socioeconomic information, including employment, migration, and disability information about the US population. These community-level indicators, which include occupation, income, and education, have been linked to a number of health outcomes, including life expectancy, self-reported health, chronic conditions, certain types of cancer, mental disorders, cardiovascular disease, obesity, and infant mortality [26]. In fact, studies have assessed the feasibility of linking patient data from HER to microdata from the ACS, with the goal of improving the understanding health disparities and social determinants of health in the population [27]. The ACS denominators generally perform comparably well and yield estimates with little bias [28]. We utilized information from the ACS as a measure for the current population to derive the incidence rates of malignant colorectal polyps. The incidence rates were calculated using a population specific to the particular year as a denominator, per 100,000 people. Because these are de-identified datasets, the study was exempted from review/approval by the Wright Center for Graduate Medical Education’s institutional review board.

### 2.2. Patient Selection

All patients diagnosed with at least a single malignant colorectal polyp on colonoscopies performed for any indication or screening between 2000 and 2017 were eligible for the analysis. All these patients were stratified according to the 6th edition of AJCC, thus ensuring uniformity in staging. We included data from patients aged between 20 and 49 years, diagnosed over the 18-year time period. Malignant colorectal polyps found during colonoscopy in the entire colon, including the rectum, were included, however, the SEER database does not differentiate data between right vs. left colon or rectum. Most of these patients underwent diagnostic colonoscopies, since screening colonoscopy began at age 50 years prior to 2018, after which, the USPSTF guidelines changed. We excluded familial cancers, lesions with a high microsatellite instability (MSI-H), and Adenomatous polyposis coli (APC).

### 2.3. Statistical Analysis

We accessed the data using the SEER diagnostic codes 8210/2 and 3 for tubular adenomas, 8221/2 for serrated polyps, 8221/2 and 3 for multiple adenomatous polyps, 8261/3 and 8262/3 for villous adenomas, and 8263/2 and 3 for tubulovillous adenomas. We performed a descriptive statistical analysis using the SPSS v27 Macintosh(SPSS v27, IBM Corporation, Armonk, NY, USA). The incidence rates per 100,000 people were calculated using data from the American Community Survey [29]. CRC incidence was stratified based on gender and histology.

## 3. Results

Between 2000 and 2017, a total of 10,742 patients with biopsy-proven sporadic malignant colorectal polyps between the ages of 20 and 49 years were included. The cohort consisted of 57.1% men and 42.9% women (Table 1).

**Figure 1:**
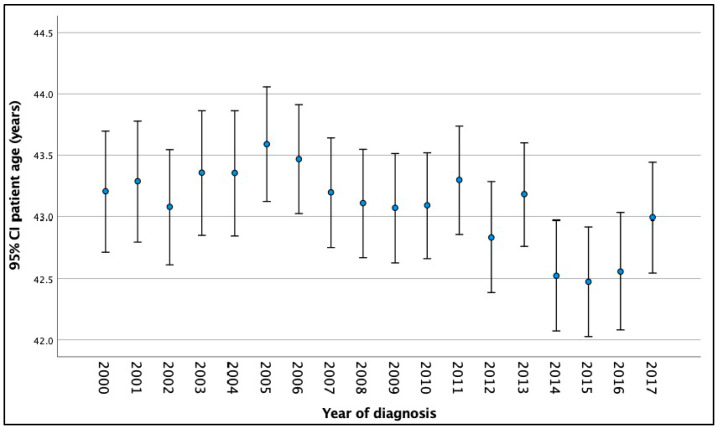
Age of malignant colorectal polyp incidence over time in 20- to 50-year age group.

The average age of malignant colorectal polyp diagnosis was 43.07 years (average age range for CRC diagnosis: 42.92–43.23 years). The annual mean age (95% CI) of new-onset malignant polyps trending over time is visualized in Figure 1. The majority of sporadic malignant polyps were diagnosed in patients aged 45–49 years, with 50% diagnosed between the ages of 45 and 49 and 25–30% diagnosed between the ages of 40 and 44.

**Figure 2:**
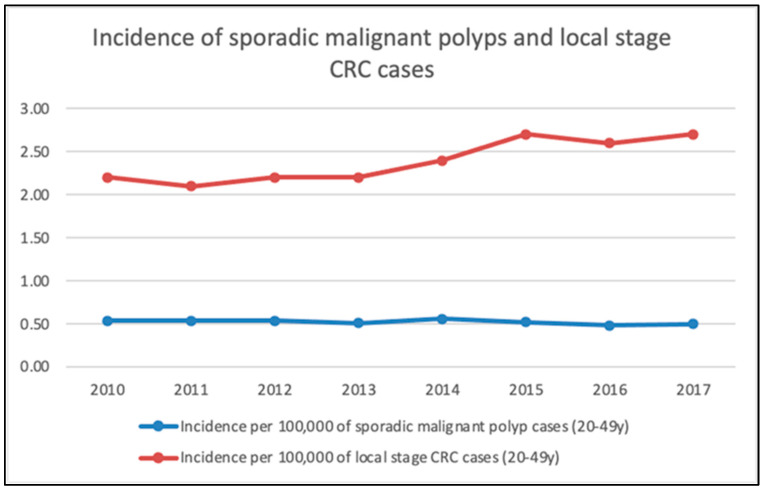
Observed incidence per 100,000 of sporadic malignant polyps and local-stage CRC cases.

The trends of incidence of sporadic malignant polyps were compared with local-stage CRC cases, which is depicted in Figure 2. SEER defines local stage as a malignancy limited to the organ of origin; no spread beyond the organ of origin; and infiltration past the basement membrane of the epithelium into the stroma of the organ. This roughly corresponds to stage T1N0M0, as per the TNM cancer staging classification for CRC. The incidence was calculated using the number of cases of sporadic malignant polyps from the SEER database and divided by the population during the same year in the same age group (20–49 years) obtained from the ACS. Thus, the incidence rate was per 100,000 people for that particular year. Since 2013, there has been a noticeable rise in cases of local-stage CRC, while the incidence of sporadic malignant colorectal polyps remained constant from 2010 to 2019 (~0.5 cases/10,000 population). Table 1 lists the histological subtypes and gender distribution of sporadic malignant colorectal polyps. There has been a significant decrease in new cases of malignant villous adenomas. However, the incidence of malignant tubular adenomatous polyps and tubulovillous adenomas has significantly increased over time. Serrated adenocarcinomas in serrated polyps were reported as very few in the population studied.

## 4. Discussion

We looked at data from colonoscopies performed on symptomatic patients across the country. The objective of the present study was to analyze the incidence and trends of young-onset CRC between 2000 and 2017. To the best of our knowledge, this is the largest known cohort to date, where we analyzed 10,742 cases of sporadic young-onset CRC over a period of 18 years across the United States.

One in five patients diagnosed with CRC under 50 years of age has a genetic predisposition syndrome [11]. There are multi-society guidelines to begin early screening in these individuals [30,31]. However, the detection of cancer among the other 80 percent of patients poses a considerable challenge, since there is no family history to advocate for early screening in this group. Conflicting guidelines exist in terms of initiating average-risk colorectal cancer screening at age 45 years. The USPSTF changed its guidelines in 2021 to recommend initiating screening at 45 years due to the increasing incidence of young-onset colorectal cancer [15]. It expanded its recommendations for CRC screening guidelines in 2021 to include adults aged 45–49 [15,18]. The ACP recently recommended not to screen average-risk individuals between 45 and 49 years [16]. This ACP guideline was based on the absence of direct evidence that screening younger individuals reduces CRC incidence or mortality [16,32].

Of note is that early experiences with screening in the 45 to 49 years group show similar rates of neoplasia as those in the 50 to 55 years group of patients [33]. Lowering the starting age of population screening for sporadic CRC to 45 years also seems to be cost-effective. Our data support screening for CRC in the younger population. The average age of CRC diagnosis was 43.07 years (range: 42.92–43.23), which is well below the ACP recommendation to start screening at 50 years. In fact, it is also lower than the USPSTF recommendation to start CRC screening at 45 years. This lack of uniformity prevents having definitive guidelines for screening in the 45 to 49 years population.

According to studies conducted around the world, the incidence of CRC is increasing in people under 50 and at a slower rate in people over 50 [34,35,36]. The total incidence of YoCRC in the United States and the number of cases of advanced-stage colorectal carcinomas increased twofold between 1990 and 2013, according to a study analyzing the SEER database [37]. While older research has established that diabetes and obesity are risk factors in the younger population [38], Austin et al. studied mean body mass index (BMI) increases across age groups, which was unable to explain the selective increase in incidence amongst younger age groups compared to the older population [39].

Our study population comprised 42.9% females, and showed increased incidence in both genders. Although there was no significant gender preponderance in our study, it was seen that, during some years, females were diagnosed with a higher number of cases of YoCRC. Our results are consistent with those of Lall et al., who found that young females are more susceptible to CRCs [40]. In contrast, some cohorts revealed a marginally higher incidence of YoCRC in men compared to women, with women consistently having fewer cases than men, while other studies found no difference between the two genders over the past three decades [37,41]. It has been studied that gender and body mass index (BMI) are associated with CRC diagnosis at a younger age, and there is a linear relationship between BMI and YoCRC [42,43]. Men are more likely than women to be diagnosed with CRC according to global trends; this has been attributed to a number of factors, including the frequency of colonoscopies, race, socioeconomic status, and insurance coverage [41,44].

The mean age at presentation and diagnosis of sporadic malignant polyps in our study was 43 years (42.9–43.23, 95% CI). As shown in Figure 1, the average age at which malignant polyps is diagnosed has decreased since 2014. The majority of malignant polyps (50%) were discovered in patients between the ages of 45 and 49, with 25 to 30 percent discovered in patients between the ages of 40 and 44. The SEER study by Wang et al. found a similar pattern, with 27% of cases diagnosed between 40 and 45 and 47% between 45 and 49 [37]. According to Abualkhair et al., 95.1% of CRCs diagnosed between the ages of 45 and 50 are invasive, with 46% increased incidence rates in 1 year age transitions [45]. The patients in our study who underwent colonoscopies had symptoms that persisted and necessitated diagnostic scopes. These lesions would have been discovered earlier had screening been performed at an earlier age.

Our analysis revealed a stable trend in overall local-stage CRCs over time, however, there has been a steady increase in the subset of sporadic malignant polyps (Figure 2). This is an interesting trend which implies that, while the number of CRCs accounted for by sporadic malignant polyps has remained steady, there has been a rise in polyps that are going undetected and progressing to local-stage CRC at presentation. This could mean an increase in the incidence of adenomas in the younger age group which progress to CRC before they are detected. Patients in the age group of 20–49 years have not been subject to CRC screening until recently, when the minimum age for cancer screening was decreased from 50 to 45 years by the USPSTF in 2019 [46]. This could also mean that there are ‘alternative’ pathways other than the traditional adenoma–carcinoma sequence of CRC which are at play. Recently, there has been increasing interest in and evidence in favor of the serrated carcinogenesis pathway [47]. Serrated polyps are the second most common type of polyp identified during a colonoscopy (after conventional adenomas). Approximately 15–30% of CRCs arise from the serrated polyp pathway. Evidence suggests that serrated polyp subtypes, especially traditional serrated adenoma (TSA) and sessile serrated adenoma/polyp (SSA/P), can cause adenocarcinoma via the serrated route. Additionally, the data indicate that SSA/Ps are the precursors of CRC through microsatellite instability (MSI) and could rapidly progress to malignancy [48]. Recent data from surveillance colonoscopies after the development of YoCRC have shown that the absence or presence of polyps is an important prognostic factor. The development of polyps during surveillance shows that it is necessary to extend the follow-up time, even in cases with microsatellite-stable YoCRC [49].

Over the course of this 18-year analysis, histopathology trends showed an increase in malignant tubular and tubulovillous adenomas and a gradual decline in malignant villous adenomas. YoCRC patients are known to be more likely to develop poorly differentiating aggressive forms of CRC, which have worse prognoses [38,50,51,52]. According to a study by Abualkhair et al. that spanned over 15 years, the incidence of localized CRC increased by 75.9%, that of regional CRC increased by 30%, and that of advanced CRC increased by 15.7% between the ages of 49 and 50 [45]. Similar research found that 90% of polyps were tubular adenomas with low-grade dysplasia, with tubulovillous adenomas with low-grade dysplasia making up 8% of these polyps [40]. Seven serrated adenocarcinomas in serrated polyps were found in our study during this period. Serrated adenomas are frequently found in the proximal colon and are challenging to see without improved colonoscopic techniques [53]. Vogelstein et al. described the pathogenesis of YoCRC to be based on an adenoma to carcinoma sequence [54]. However, this cannot explain genetic etiologies of CRC, as these appear phenotypically different from old CRC [55]. Changes in genotypic driver mutations in the adenoma–carcinoma sequence have been linked to phenotypic profile changes found in YoCRC. Many genes identified to have caused mutations in YoCRC are not commonly seen in the presumed adenoma–carcinoma sequencing. Further research should focus on younger patients with sporadic malignant colorectal polyps [56,57].

It is important to consider the fact that gastrointestinal diseases are responsible for considerable healthcare use and expenditure. In 2018 alone, gastrointestinal healthcare expenditures totaled USD 119.6 billion in the United States [58]. Increasing awareness about early CRC detection through regular screening is an effective strategy to reduce the economic burden of CRC [59]. In the past 5 years, the USPTF, ACG, and ACS updated their screening recommendations for CRC from the age of 50 to start at the age of 45 years [17,18,19]. The biggest challenge in the near future will be to improve screening in the newly eligible, those overdue and unscreened, and reduce barriers to cancer care [32]. Since this modification to standard CRC screening, there have not been enough studies looking at the prevalence of YoCRC. Our study examined data collected prior to 2018, when screening recommendations changed and colonoscopies were performed in response to symptomatic presentations. We noticed a significant increase in the incidence of YoCRC in patients aged 45–49, as well as those aged 40–45 years. It is known that these polyps might have existed years before the age of screening; if these cases had been screened for earlier, the majority of diagnoses of YoCRC might have been avoided [60]. We also looked at changes in the histopathological trends of young-onset malignant polyps, which are probably the result of evolving genotypic mutations. Additionally, there is a need for more studies to better understand the pathogenesis and evolution of YoCRC.

Among our study’s strengths is that SEER remains widely regarded as the gold standard for data quality among cancer registries in the United States and around the world. We leveraged the strengths of the SEER program with regard to representativeness and generalizability to the U.S. population, the lengthy period of data collection, the large numbers of cases, and the collection of cancer specific outcomes. The registry includes patients treated in a wide range of practice settings and currently represents 42% of the US population. SEER’s stringent protocols and quality control ensure data reliability and accuracy. The SEER database was used to provide epidemiological trends in sporadic malignant polyps of young onset in our study. We calculated the incidence per 100,000 people in the United States to provide a standardized denominator for comparison.

Since this is a population-based retrospective study, it has some inherent limitations. The SEER database has an inclusion bias, with limited staging and metastatic data recorded. Because the database only collects malignant data, we were unable to include any pre-malignant lesions. SEER only reports first treatment interventions. As a result, there was no information on the subsequent recurrence of malignant polyps. However, recurrence data should not be used to calculate the incidence of primary sporadic malignant polyps. There is also a lack of information on comorbidities. Furthermore, detailed information regarding treatment and prognosis is lacking. Nonetheless, the study remains convincing, given the large demographics.

## 5. Conclusions

Malignant sporadic polyps account for one-quarter of all localized CRCs diagnosed in people under the age of 50. Significant upward trends in sporadic malignant polyps were observed over time, possibly reflecting changes in tumor biology, necessitating further research. The majority of these sporadic malignant polyps were diagnosed between 40 and 49 years. These findings may have potential implications for future CRC screening strategies in younger patients. Improving screening in the newly eligible population within a framework of health equity and reducing barriers to care remains important to further reduce the burden of CRC.

## Figures and Tables

**Table 1:** Gender distribution and histological trend over time (2000–2017) of malignant colorectal polyps in patients aged 20–49 years.

	2000	2001	2002	2003	2004	2005	2006	2007	2008	2009	2010	2011	2012	2013	2014	2015	2016	2017
**Female**	226	235	242	242	223	235	282	307	293	329	355	315	346	332	342	315	332	340
**Male**	245	233	266	250	247	268	286	291	356	349	325	365	341	316	379	356	389	306
**Adenocarcinoma in adenomatous polyp**	169	152	191	166	184	179	239	237	245	270	247	283	281	276	309	298	280	275
**Serrated adenocarcinoma**	0	0	0	0	0	1	0	0	1	0	0	0	0	2	0	0	1	2
**Adenocarcinoma in multiple adenomatous polyp**	1	3	2	4	3	6	1	3	1	0	2	1	3	4	2	0	1	0
**Adenocarcinoma in villous adenoma**	108	112	101	101	91	95	93	101	101	96	91	79	72	61	64	59	49	46
**Adenocarcinoma in tubulovillous adenoma**	193	212	214	221	192	222	244	257	301	312	340	317	331	305	346	314	390	323

## Data Availability

The data that support the findings of this study are openly available within the Surveillance Epidemiology and End Results (SEER) database at http://seer.cancer.gov/ (accessed on 21 October 2023). NIH. National Cancer Institute: Surveillance, Epidemiology and End results (SEER) program. Available online: https://seer.cancer.gov/ (accessed on 21 October 2023). The Registry of Research Data Repositories persistent identifier for this resource is RRID: nif-0000-21366.
